# Enriched Astaxanthin Extract from *Haematococcus* *pluvialis* Augments Growth Factor Secretions to Increase Cell Proliferation and Induces MMP1 Degradation to Enhance Collagen Production in Human Dermal Fibroblasts

**DOI:** 10.3390/ijms17060955

**Published:** 2016-06-16

**Authors:** Hsin-Yu Chou, Chelsea Lee, Jian-Liang Pan, Zhi-Hong Wen, Shu-Hung Huang, Chi-Wei John Lan, Wang-Ta Liu, Tzyh-Chyuan Hour, You-Cheng Hseu, Byeong Hee Hwang, Kuo-Chen Cheng, Hui-Min David Wang

**Affiliations:** 1 Department of Fragrance and Cosmetic Science, Kaohsiung Medical University, Kaohsiung 807, Taiwan; s9412105@yahoo.com.tw; 2 Division of Biochemistry and Molecular Biology, Graduate Institute of Medicine, College of Medicine, Kaohsiung Medical University, Kaohsiung 807, Taiwan; cliff@cc.kmu.edu.tw; 3 Department of Psychology, Michigan State University, East Lansing, MI 48824, USA; chejlee@umich.edu; 4 Department of Applied Cosmetology, Kao Yuan University, Kaohsiung 821, Taiwan; t50050@cc.kyu.edu.tw; 5 Degree Program of Cosmetology and Health Care, Kao Yuan University, Kaohsiung 821, Taiwan; 6 Department of Marine Biotechnology and Resources, National Sun Yat-sen University, Kaohsiung 804, Taiwan; wzh@mail.nsysu.edu.tw; 7 Graduate Institute of Medicine, College of Medicine, Kaohsiung Medical University, Kaohsiung 807, Taiwan; huangsh63@gmail.com; 8 Division of Plastic Surgery, Department of Surgery, Kaohsiung Medical University Hospital, Kaohsiung Medical University, Kaohsiung 807, Taiwan; 9 Department of Surgery, Faculty of Medicine, College of Medicine, Kaohsiung Medical University, Kaohsiung 807, Taiwan; 10 Center for Stem Cell Research, Kaohsiung Medical University, Kaohsiung 807, Taiwan; 11 Biorefinery & Bioprocess Engineering Laboratory, Department of Chemical Engineering and Materials Science, Yuan Ze University, Chung-Li 320, Taiwan; lanchiwei@saturn.yzu.edu.tw; 12 Department of Biotechnology, Kaohsiung Medical University, Kaohsiung 807, Taiwan; liuwangta@kmu.edu.tw; 13 Department of Biochemistry, School of Medicine, Kaohsiung Medical University, Kaohsiung 807, Taiwan; 14 Department of Cosmeceutics, College of Biopharmaceutical and Food Sciences, China Medical University, Taichung 40402, Taiwan; ychseu@mail.cmu.edu.tw; 15 Department of Health and Nutrition Biotechnology, Asia University, Taichung 41354, Taiwan; 16 Division of Bioengineering, Incheon National University, Incheon 22012, Korea; bhwang@inu.ac.kr; 17 Department of Internal Medicine, Chi-Mei Medical Center, Tainan 710, Taiwan; 18 Department of Safety Health and Environment, Chung Hwa University of Medical Technology, Tainan 717, Taiwan; 19 Department of Medicine, National Defense Medical Center, Taipei 114, Taiwan; 20 Graduate Institute of Natural Products, Kaohsiung Medical University, Kaohsiung 807, Taiwan; 21 Center for Infectious Disease and Cancer Research, Kaohsiung Medical University, Kaohsiung 807, Taiwan

**Keywords:** enriched astaxanthin extract (EAE), *Haematococcuspluvialis*, phorbol 12-myristate 13-acetate (PMA), doxycycline

## Abstract

Among many antioxidants that are used for the repairing of oxidative stress induced skin damages, we identified the enriched astaxanthin extract (EAE) from *Haematococcus* *pluvialis* as a viable ingredient. EAE was extracted from the red microalgae through supercritical fluid carbon dioxide extraction. To compare the effectiveness, EAE wastreated on human dermal fibroblasts with other components, phorbol 12-myristate 13-acetate (PMA), and doxycycline. With sirius red staining and quantitative real-time polymerase chain reaction (qRT-PCR), we found that PMA decreased the collagen concentration and production while overall the addition of doxycycline and EAE increased the collagen concentration in a trial experiments. EAE increased collagen contents through inhibited *MMP1* and *MMP3* mRNA expression and induced *TIMP1*, the antagonists of MMPs protein, gene expression. As for when tested for various proteins through western blotting, it was seen that the addition of EAE increased the expression of certain proteins that promote cell proliferation. Testing those previous solutions using growth factor assay, it was noticeable that EAE had a positive impact on cell proliferation and vascular endothelial growth factor (VEGF) than doxycycline, indicating that it was a better alternative treatment for collagen production. To sum up, the data confirmed the possible applications as medical cosmetology agentsand food supplements.

## 1. Introduction

Skin, the largest organ of the human body, functions as our body’s protective shield against exterior factors whether they are physical, chemical, or biological [1,2,3]. Skin is essential in the protection of the body from damages and for wound repair [4,5]. These multiple skin layers work together to barricade the internal components from chemicals, extreme temperatures, and damaging sunlight consisting ultraviolet rays [6]. The sun radiates visible light, infrared radiation interpreted as heat, and ultraviolet ray which causes oxidative stress to the human skin [7]. In reactive oxygen species (ROS) biology, superoxide, was identified as the major ROS species induced by phorbol 12-myristate 13-acetate (PMA) but not by ionomycin in mouse macrophages. Thus, PMA has been routinely used as an inducer for endogenous superoxide production [8]. It may cause cancerous benign or malignant tumors after long, repeated exposures sunlight. Reducing oxidative stress isa good way to keep human being physiologically healthy.Therefore, antioxidants from natural plant species decrease oxidative stress from external and intrinsic sources have many applicable biofunctions keeping human skin care [9,10].

The matrix metalloproteinase (MMP) consist of over 20 zinc peptidases of the metzincin super-family in human [11,12]. This series of enzymes is responsible for the maintenance and turnover of macromolecules of extracellular matrix (ECM), like collagen. MMP1 is a critical initiator of ECM exacerbation and associated with other MMPs in collagen degradation. MMP1 expression from human dermal fibroblasts is induced by some physiological factors, including inflammatory cytokines, growth factors, tumor promoters, and ultraviolet radiation. PMA is recognized with the contribution to MMP1 production through the activation of c-Jun, extracellular signal-regulated kinases (ERK) and inhibiting the tissue inhibitor of metalloproteinases-1 (TIMP1) [13]. Remodeling and synthesis of ECM collagen in human dermal fibroblasts are important to cell proliferation. High cell growth and migration of fibroblasts prompt the wound healing progressions. Vascular endothelial growth factor (VEGF) [14,15], is a critical factor participating in the skin cell proliferation. Skin cells are settled in the ECM, which belongs to the connective tissue, provides structural support, and regulates cell communications and behaviors. Collagen is the main component of ECM and is the most bountiful protein in connective tissue.

Astaxanthin has a chemical formula of C_40_H_52_O_4_ and a molecular weight of 596.86 in geometric *cis*- and *trans*-isomers; the latter is thermodynamically more stable than the former. It is found predominantly in nature, and it possesses potent antioxidant activity, which has been demonstrated in numerous studies. Astaxanthin is much more effective in scavenging free radicals than other carotenoids and Vitamin E. It is the powerful anti-oxidative activity that makes astaxanthin beneficial to human health. Enriched astaxanthin extract (EAE) was extracted from the red microalgae, *Haematococcus pluvialis*, through supercritical fluid carbon dioxide extraction (SFE-CO_2_) to elucidate the possible role of viable food and cosmetic ingredients to enhance the proliferation of skin cell. We examined the protective effect of EAE on PMA induced reactive nitrogen/oxygen species production and proteins in cultured fibroblasts.

## 2. Results

### 2.1. 1,1-Diphenyl-2-picrylhydrazyl (DPPH) Free Radical Scavenging Activity Assay

We carried out the radical scavenging assay using 1,1-Diphenyl-2-Picrylhydrazyl (DPPH). DPPH is an antioxidant assay to detect antioxidants scavenging free radical ability. Therefore, this method examination has been used for antioxidant assessment broadly. To investigate the antioxidant activities of EAE, a dosage of 10 μg/mL was used to determine the scavenging properties, respectively. In Table 1, we illustrated that EAE had moderate inhibition value (45.3%) in DPPH scavenging assay while vitamin C at the same condition, 100 μM (86.5%).

### 2.2. Ferrous Ions Chelating Capacity

The ferrous ion chelating activity of EAE was described in Table 1. In ferrous ion chelating activity assay, the Ethylenediaminetetraacetic acid (EDTA) at 100 μM similar to our EAE, and EDTA was included as a positive control. With the existence of chelating mediators, the Fe^2+^ complex formation was broken, resulting in a reduction, ferrous ions, from a dark red color of the complex. EAE at the dosage of 10 μg/mL presented minor levels on Fe^2+^ scavenging effectiveness of 54.7%, respectively. EDTA obsessed ion chelating ability of 87.3% at 100 μM.

### 2.3. Reducing Power

Ferric reducing potential assay is another simple and trusty analysis. Ferric reducing potential system is used to quantify the reducing ability of an antioxidant reacting with a ferric 2,4,6-tripyridyl-*S*-triazine Fe(III) (TPTZ) complex which produces ferrous Fe(II)-TPTZ complex with a dark blue color by an adopted reductant. In Table 1, EAE presented a slightly higher ferric reducing power.

### 2.4. Cell Growth of Enriched Astaxanthin Extract (EAE) Treated in Human Fibroblasts

**Figure 1:**
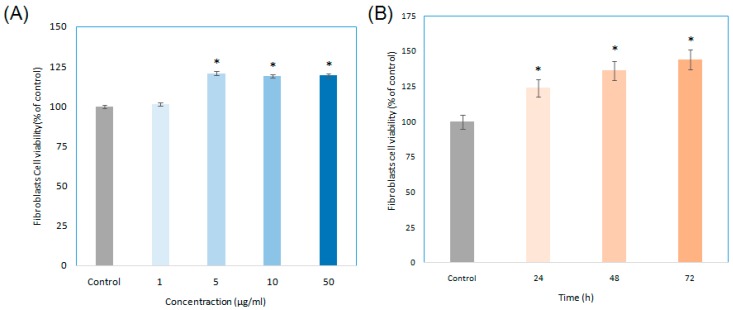
Enriched astaxanthin extract (EAE) effects on human cell viability with various doses and time intervals. Human skin fibroblasts were seeded in a 96-well micro titer plate which had a density of about 1 × 10^4^ cells/well and (**A**) treated with 1, 5, 10, and 500 μg/mL of EAE for 24 h; at 0 μg/mL as the control group; (**B**) treated with 50 μg/mL for 24, 48 and 72 h; at 0 h as the control group. The cell viability of fibroblasts were measured by 3-(4,5-Dimethylthiazol-2-yl)-2,5-diphenyltetrazolium bromide (MTT assay) 24 h after compound treatment. (Data represents mean ± S.D. of three independent experiments performed. * *p* < 0.01).

By SFE-CO_2_, we were able to obtain various concentrations of EAE from the red microalgae *H. pluvialis*. As shown in Figure 1A, we found that EAE yielded cell growths at various doses (0–50 μg/mL). The average cell viability when time was the independent factor for all the varying concentrations of EAE was calculated at 24, 48, and 72 h, and found to be 123.90%, 136.32%, and 143.98% in the same order. This indicated that the concentration of EAE will not effect the viability of the fibroblasts compared to the control trial after the addition of EAE into the medium. As for how the length of time after EAE was added to the medium effect the cell viability of the fibroblasts, we looked towards Figure 1B for indications. As the chart represents, as the length of time after the addition of EAE into the medium increased, the cell viability of fibroblasts enhanced as well.

### 2.5. Effects of EAE on Phorbol 12-Myristate 13-Acetate (PMA)-Stimulated MMP1 and TIMP1 Production in Human Fibroblasts

**Figure 2:**
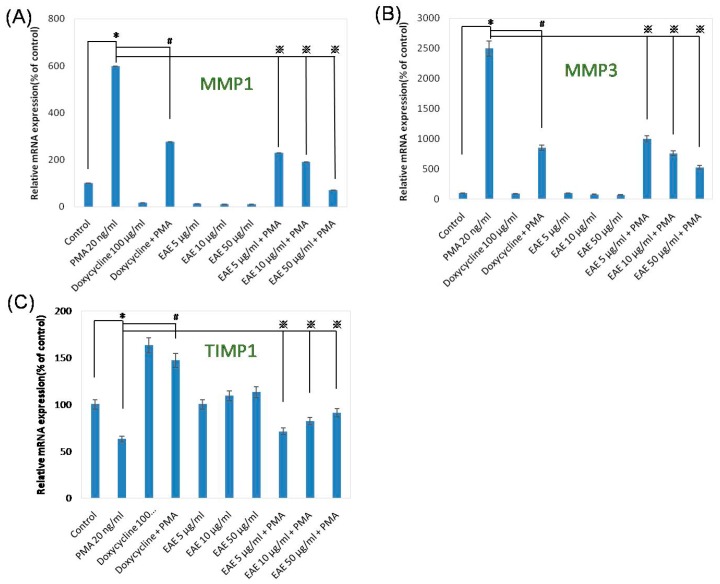
EAE decreased mRNA expressions MMP1 and MMP3, enhanced TIMP1 mRNA production in human noral epidermal fibroblasts at 24 h. (**A**) MMP1; (**B**) MMP3; (**C**) TIMP1. Phorbol 12-myristate 13-acetate (PMA) was at 20 ng/mL as the negative control, and doxycycline was at 100 μg/mL as the positive control within this assessment (Data represents mean ± S.D. of three independent experiments performed. * *p* < 0.01, # *p* < 0.01 and ※ *p* < 0.01).

When we were studying how MMP1 was affected, the range in relative mRNA expression due to the effect of varying components compared to the control was very large (Figure 2A). The largest difference was found when comparing the control to the addition of 20 ng/mL of PMA, with a +498% difference. As for when doxycycline was introduced in a different trial, MMP1 expression was greatly decreased by 83%. Even when doxycycline was added along with PMA into the solution, it decreased the enzyme expression compared to if only the compound contains PMA with a difference of −322%. EAE also affected MMP1 similarly to doxycycline, but to a larger extent. When only EAE was a component, even at the lowest dose of 5 µg/mL, the mRNA expression of MMP1 was lower than if only doxycycline was inserted, a contrast of −5%. It was comparable to the addition of PMA and doxycycline to the solution, when EAE and PMA were introduced together in one scenario; MMP1 expression was lower than if the addition only contains PMA. The range of difference between PMA and EAE with PMA was between −368% and −528% among the different EAE concentrations tested. There was a parallel trend among doxycycline with PMA and EAE with PMA, with the latter of the two showing a larger difference compared to the solution containing only PMA. As we saw in the testing of just doxycycline or EAE, doxycycline had a lesser effect on MMP1 compared to the enriched astaxanthin extract but both decrease the expression of MMP1 which broke down collagen. MMP3 had alike trends to what was observed for MMP1 (Figure 2A,B). The addition of PMA increased the mRNA expression while doxycycline and EAE decreased the relative expression. TIMP1 was also different from the previous two proteins in that as the concentration of EAE increases, the mRNA expression increases. In the experimentation with MMP1 and MMP3, the expression of these two proteins decreased as the concentration of EAE increases. As for the influences that the additions had on TIMP1 mRNA expression, they were not as drastic as the other two enzymes previously stated (Figure 2). The EAE also worked in an inverse relationship for TIMP1 compared to MMP1 and MMP3, which could be explained since TIMP1 allowed for collagen growth while MMP1 and MMP3 induced collagen breakdown. When the solution comprised of 20 ng/mL of PMA, it decreased the relative expression of TIMP1 by −37%.

**Figure 3:**
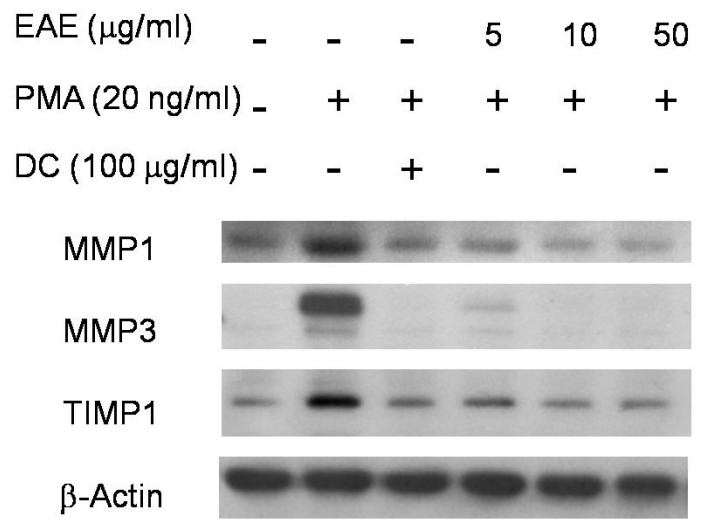
Western blotting was performed to compare cellular protein expressions. EAE altered the collagen-related protein levels. PMA was at 20 ng/mL as the negative control, and doxycyline (DC) was at 100 μg/mL as the positive control.

To further showcase how EAE influenced the mRNA expressions of certain proteins, the same tested solution compounds in the human dermal fibroblast were used to study the mRNA expressions of other proteins (Figure 3). PMA, being a protein that discouraged collagen production, decreased the expression of TIMP1. When PMA and doxycycline were added, the expression of TIMP1 does increased, but not as significantly as when EAE was substituted in for doxycycline. As for MMP1 and MMP3, which had alike functions of breaking down collagen, they showed a significant decrease in gene expression from western blotting with the marker being barely visible when EAE was added to the medium along with PMA. When only PMA was added to the medium though, there was a substantial increase in gene expression for MMP1 and MMP3, signifying that these proteins had higher expressions within the fibroblasts.

### 2.6. Collagen Productions in Sirius Red Assays

**Figure 4:**
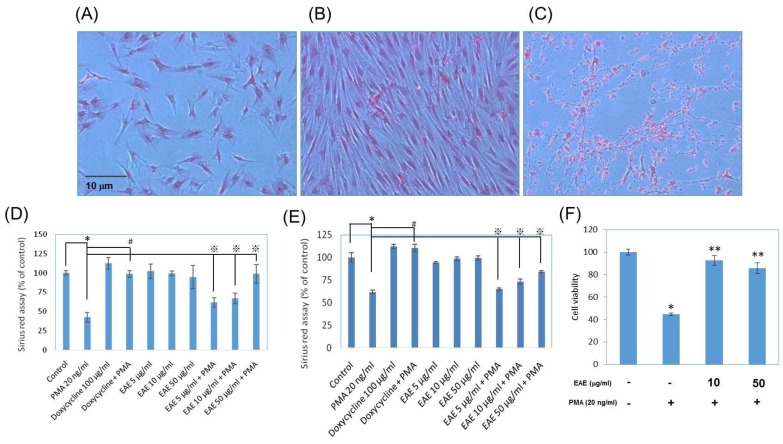
Human normal epidermal fibroblasts collagen production with EAE treatments in sirius red assay. In (**A**) Day 1, at 50 μg/mL; (**B**) Day 7, at 50 μg/mL; and (**C**) PMA, at 20 ng/mL treated 24 h. Sirius emitted bright red color (microscopy, the magnification is 100×); (**D**) Day 1 and (**E**) Day 7 have been quantitative; (**F**) Anti-oxidative effect of EAE in PMA-activated fibroblast viability. (Data represents mean ± S.D. of three independent experiments performed. * *p* < 0.01, # *p* < 0.01 and ※ *p* < 0.01).

As evident in Figure 4A,B, a typical healthy cell that has had no additional compounds within the solution increased in collagen after the seven days of experimentation. Looking at Figure 4A,C, it was shown that in a solution containing PMA, there was a decrease in collagen after only 20 h. The graph below was indicated that on day 1 (Figure 4D), PMA decreased the collagen amount the most out of all the test compounds. EAE for all concentrations and all of the varying concentrations of EAE along with PMA also had less collagen production than the vehicle control. The trials with doxycycline were the only ones that increased the collagen production on day 1. After allowing the cells to incubate after seven days, the ones that had less collagen produced than expected according to the comparison with the control included the medium with PMA, doxycycline with PMA, and all of the varying concentrations of EAE along with PMA (Figure 4E). PMA induced oxidative stress to make human fibroblast cell death, and after treated with EAE in different concentrations (10 and 50 μg/mL), the cellular viability repaired to 95.2% and 93.3% (Figure 4F).

### 2.7. Vascular Endothelial Growth Factor (VEGF) Secretions from Human Fibroblasts

**Figure 5:**
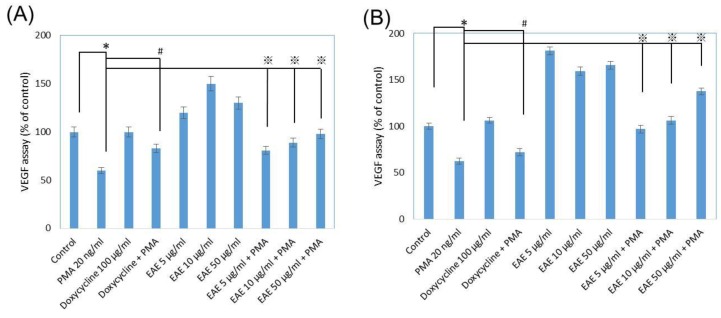
Human normal epidermal vascular endothelial growth factor (VEGF) production were in fibroblasts with EAE treatments after (**A**) Day 1 and (**B**) Day 7 of incubation. PMA at 20 ng/mL as the negative control, and doxycyline at 100 μg/ mL as the positive control (Data represents mean ± S.D. of three independent experiments performed. * *p* < 0.01, # *p* < 0.01 and ※ *p* < 0.01).

Figure 5A,B indicated the difference in VEGF production on day one compared to day seven while human dermal fibroblasts were suspended in the different solutions mentioned previously. With the addition of EAE at all different concentrations, we discovered that on day one, the increase of EAE only ranged between +20% and +33%. As for day seven, VEGF production increase due to EAE ranged between +51.25% and +65.625%, having a much larger increase in VEGF after incubating the cells in EAE than the control group was able to after the week. Varying concentrations of EAE along with PMA also was able to increase VEGF secretion on the seventh day when observed. On day 1, it had previously stated that VEGF amount of the cells in these three different concentrations of EAE with PMA was less than the vehicle control group. Then, on day seven, VEGF growth was above the control, indicating EAE reversed the consequence that PMA usually had on VEGF. PMA even after the seven days of incubation still had much less VEGF production compared to the control trial. This VEGF assay indicated that EAE had the capability to reverse the impact that PMA and ultimately, MMP1 had on skin cells, allowing for VEGF growth to permit more collagen growth for tissue repair.

## 3. Discussion

Astaxanthin is a xanthophyll carotenoid found in marine seafood and plants such as salmon, lobster, shrimp and crab, and its natural red color is responsible for brightening the flesh, skin, or exoskeleton of these animals. As a potent scavenger of free radicals and quencher of reactive oxygen and nitrogen species, astaxanthin is an effective antioxidant demonstrating greater potency than carotene carotenoids [16,17,18]. Using SFE-CO_2_ has allowed the examination that indeed EAE concentration to a certain amount from our experiment, it was found to be low concentration has a profound immediate impact on human dermal fibroblast viability. As the observation continued for up to 72 h after the skin cells were exposed to the varying concentrations of EAE, the average of all the varying concentrations indicated that the cell viability increased shown by the positive trend.

Besides examining the effects that EAE has on the overall human dermal fibroblasts, it was also used for even more detailed investigations on the impact it has MMP expressions. To understand the difference EAE effects were from varying compounds such as PMA and doxycycline, as well as the combination of these compounds were also tested as a component of the solution. Both MMP1 and MMP3, when expressed, play a role in collagen breakdown while TIMP1 expression indicates collagen production [19]. EAE slightly increased the gene expression for TIMP1 compared to the control trial which had a role in cell regulation or growth of cells. Comparing two pairs of doxycycline and PMA with EAE and PMA, the former group had more impacts, seen with the fainter marker of the proteins than the later group. In protein analysis, actin, being a protein that was not involved with collagen, and did not show any effects with PMA or EAE addition, indicating that actin had no involvements with other proteins [20,21]. The ones of the varying concentrations of EAE in addition with PMA had increased in comparison with the control group though, being closer to the number we would expect. The trials that were able to successfully increase the amount of collagen produced after a week’s time include doxycycline and all of the varying EAE concentrations, all being above 100% which was what our comparison was at. Though doxycycline had increased after the seven days of incubation, it had originally started with more than the control, unlike the varying concentrations of EAE which originated with less than 100% of collagen. The data indicated that EAE had stronger cell growth ability than doxycycline, being able to go from less than the control to proliferate more collagen than the control group after the seven days.

The enriched astaxanthin extract taken from *H. pluvialis* has proven time and time again to be a viable treatment for collagen production, and ultimately the reducing of MMP1 and MMP3 expression which would otherwise be used in collagen breakdown. EAE has been seen to have an increase in cell viability after a length of time, not just only having an immediate impact on the cell in question. The decrease in mRNA expression for MMP1 and MMP3 as well as the increase of TIMP1 which functions for collage production is another indication that EAE allows for more collagen production while decreasing the enzymes needed for collagen breakdown. Through Sirius Red assay and VEGF assay, we can see from the data gathered that collagen does grow with the addition of EAE due to the growth of VEGF that is a factor in cell growth. These examinations of EAE in comparison with an alternative doxycycline and an antagonist PMA give a strong indication that enriched astaxanthin extract from *H. pluvialis* is a positive alternative treatment for skin damage compared to others.

## 4. Experimental Section

### 4.1. Supercritical Fluid Carbon Dioxide Extraction (SFE-CO_2_)

*H. pluvialis* biomass was obtained from the Kao Yuan University (Kaoshiung, Taiwan) cooperative research project. The equipment for SFE-CO_2_ was designed by the author and produced by JeoouRong Industrial Co., Ltd. (Kaoshiung, Taiwan) [22]. The equipment is composed of a 300 mL extraction tank with a container (60 mm in diameter), which was covered by porous foaming stainless steel on both sides to prevent the extract from being taken away by fluid, causing blocking of the channel. The temperature of the liquid carbon dioxide was first reduced, with a bath tank, down to −4 °C, increased pressure with a pump to the supercritical level, went through the set thermostatic water bath (the temperature setting was the same as the extraction tank), and finally entered the extraction tank. The extraction pressure was controlled by a needle valve and a micron metering valve while the extraction temperature was controlled by a heating module. The carbon dioxide flow rate was regulated by a float flowmeter, and the accumulation of carbon dioxide volume was recorded by a wet-type gas meter. For each batch operation, the micron metering valve was first closed and when the extraction tank reached the set temperature, the container was taken out to load the *H. pluvialis* biomass and was then placed back into the original position. The ethanol modifier was pumped into the tube during the extraction and evenly mixed with the supercritical carbon dioxide fluid by a fluid mixer to pass through the thermostatic water bath with the same temperature setting as the extraction tank before entering the tank. The mixed fluid went through the *H. pluvialis* biomass and extracted the antioxidants till the set static extraction time. After that, the fluid flew through the separation tank, micron metering valve, and wet-type gas meter to the air. When the set dynamic extraction time was reached, the extraction was stopped and the extracts were collected to analyze the antioxidant contents. The emitted carbon dioxide was further collected for cultivating microalgae to reduce the environmental impact and to reduce carbon dioxide emission. The optimal operation parameters for SFE-CO_2_ of astaxanthin from *H. pluvialis* were 21.67 g/L (the weight of *H. pluvialis* biomass per the volume of extraction vessel, *w*_t_), 6.0 NL/min (CO_2_-flow rate, *f*_r_), 20.0 min (extraction time, *t*), 4500 psi (extraction pressure, *P*), 9.23 mL/g (the volume of ethanol modifier per the weight of *H. pluvialis* biomass, *V*_E_), 50.0 °C (extraction temperature, *T*) and 99.5% (modifier composition, *E*_C_).

### 4.2. Saponification of Astaxanthin Esters

Saponification was carried out to hydrolyze astaxanthin esters following a modified method described by Pan *et al.* [23]. In general, saponification was conducted by passing nitrogen (N_2_) through a mixture consisting of 5.0 mL of extraction fluid, 15.0 mL of methanol, and 6.0 mL of saponification solution (3.5 M NaOH) at 15 °C for 24 h. The process was carried out in darkness, and the nitrogen supply was cut off when the volume was reduced to 10.0 mL, and the saponification process was complete. The resulting fluid was then kept in the dark at −21 °C for analysis. To enhance the astaxanthin yield, it is necessary to hydrolyze astaxanthin esters present in the *H. pluvialis* cells via saponification process with the addition of NaOH. The saponification index of the original extracted sample was 1.0 but it increased to 12.78 by saponification with the optimal 3.5 M NaOH.

### 4.3. Determination of 1,1-Diphenyl-2-picrylhydrazyl (DPPH) Radical Scavenging Capacity

DPPH is an antioxidant assay to detect antioxidants scavenging free radical ability. It is a purple chemical reagent with stable free radial, and it will change to bright yellow if DPPH solution reach could scavenge free radical compounds [4]. Correct concentrations of the EAE were added to DPPH (60 μM) solution. After hydrogen of DPPH radicals transfer to anti-oxidative agents, the color of DPPH solution becomes light color at 517 nm resulting from the reduction in optical absorbance. The percentages of remaining DPPH were plotted against the sample to obtain the amount of antioxidant required to reduce the initial concentration of DPPH. Scavenging activity (%) was determined as

(1) $$\text{Scavenging activity }(\%)=\;\frac{\left(A_{\text{control}}-A_{\text{sample}}\right)}{A_{\text{control}}}\;\times \;100\%$$

### 4.4. Metal Chelating Activity

The ferrous ion chelating power of EAE was tested based on an earlier portrayed assay [4]. Briefly, EAE was loaded into 10 μL FeCl_2_·4H_2_O (2 mM) then added 20 μL ferrozine (5 mM); the mixture was shaken, and retained to stand for 10 min at 25 °C. The absorbance of the testing solution was observed at 562 nm. EDTA was used as a positive control, and the chelating power calculation formula was accorded to Equation (1).

### 4.5. Reducing Power

The reducing property of EAE was according to the Oyaizu [4]. In Brief, EAE was added to 85 μL phosphate-buffered saline (PBS) buffer (67 mM, pH 6.8), K_3_Fe(CN)_6_, 2.5 μL and 20% potassium ferricyanide. Then reacted for 20 min at 50 °C, and following 160 μL of trichloroacetic acid (10%) was mixed to the reactant to centrifuge at 3000× *g* for 10 min. The solution mixed with 2% FeCl_3_ (25 μL), and the optical density was read at 700 nm via a 96-well plate. We used Butylated hydroxyanisole (BHA) as a positive control, and higher optical absorbance means a higher reductive property.

### 4.6. Cell Cultures

Neonatal foreskin primary human epidermal fibroblasts were purchased from Cascade Biologics™ (Portland, OR, USA), cultured in Medium 254 (M-254-500; Cascade Biologics™), and supplemented with human melanocyte growth supplement (HMGS, Cascade Biologics™).The Medium 254 is a basal medium containing essential and non-essentialamino acids, vitamins, organic compounds, traceminerals, and inorganic salts. The human melanocyte growth supplement contains bovine pituitary extract, fetal bovine serum, bovine insulin, bovine transferrin, basic fibroblast growth factor, hydrocortisone, heparin, and PMA. Cells were incubated at 37 °C in a humidified incubator 5% CO_2_ atmosphere. To study melanin biogenesis, HEMn-MP were incubated in 24 well-plates at a density of 10^5^ cells per well.

### 4.7. Cell Proliferation Examinations

Human dermal fibroblasts were placed in a consistent monolayer culture of Dulbecco’s modified Eagle medium (DMEM) for a time period of 24, 48, or 72 h with 5% CO_2_ and 37 °C [24]. The DMEM also contained 10% fetal bovine serum (FBS), 100 U/mL of penicillin, 100 mg/mL of streptomycin, and 0.25 µg/mL of amphotericin B. Enriched astaxanthin extract (EAE) acquired from *Haematococcus pluvialis*, PMA, and doxycycline were dissolved either by themselves or in addition without another component in dimethyl sulfoxide (DMSO) at varying concentrations, ending with the concentration of DMSO to be less than 1%.

### 4.8. Quantitative Real Time Polymerase Chain Reactions

To measure the mRNA expression of the varying proteins that were going to be experimented on MMP1, MMP3, and TIMP1 quantitative real time polymerase chain Reaction (qRT-PCR) was used. 20 µL of a reaction that includes 10 µL of 2× Quantitect SYBR Green Mast Mix (Qiagen, Valencia, CA, USA) contained hot start Taq polymerase, 0.4 mL mix of two reverse transcriptases, 0.5 mL (10 ng/mL) of template, and 0.8 mL of primers. The primer sequence used for MMP1 was forward 5′-CCCTCTTGAACTCACATGTTATG-3′ and reverse 5′-ACTTTCCTCCCCTTATGGATTCC-3′. As for the MMP3 primer, the forward sequence was 5′-TCCTCATATCAATGTGGCCAAA-3′ and 5′-CGGCACCTGGCCTAAAGAC-3′ for the reverse sequence. Lastly, the TIMP1 primer sequences used were 5′-CACCAGAGAACCCACCATGGC-3′ (forward), 5′-CACTCTGCAGTTTGCAGG-3′ (reverse). To conduct real-time PCR assays, the StepOnePlus™ System (Thermo Fisher Scientific, Portland, OR, USA) was used as well as the corresponding instructions to carry out the reaction. According to the manual, the RT reaction was run for 20 min at 42 °C, 5 min at 95 °C for the FastStartTaq DNA polymerase to become activated, and another 40 or 50 cycles of amplification. The amplification consisted of 5 s is needed at a temperature of 95 °C for denaturation, another 5 s at 60 °C for annealing and acquisition, and finally elongation at 72 °C for 15 s. Fluorescence acquisition was done at the end of the annealing phase in this particular experiment but it can also be conducted at the end of the elongation phase.

### 4.9. Western Blotting

The human dermal fibroblasts used in this experiment were treated with 20 ng/mL of PMA, 100 µg/mL of doxycycline, PMA along with doxycycline, EAE at 5, 10, and 50 µg/mL, PMA in addition with the varying concentrations of EAE mentioned previously, or the control medium for 24 h. The skin cells were then harvested and lysed with the use of lysis buffer (50 mM Tris-HCl, pH 7.5, 137 mM sodium chloride, 1 mM EDTA, 1% Nonidet P-40, 10% glycerol, 0.1 mM sodium orthovanadate, 10 mM sodium pyrophosphate, 20 mM β-glycerophosphate, 50 mM sodium fluoride, 1 mM phenylmethylsulfonyl fluoride, 2 μM leupeptin and 2 μg/mL aprotinin). The lysates that were harvested were then centrifuged for 30 min at 12,000 rpm. The protein concentration found in the supernatant after centrifuging was determined by the use of bicinchoninic acid (BCA) protein assay kit (Pierce, Rockford, IL, USA). The proteins being examined from each sample were taken in equal quantities and resolved by sodium dodecyl sulfate-polyacrylamide gell electrophoresis (SDS-PAGE) on 12% gel and transferred electrophoretically to a nitrocellulose membrane [24]. Carefully, the transfer film was removed from the wet transfer tank and semidry transfer slot, it was placed in the box containing 5% skim milk prepared for one hour at room temperature in 1× TBST (Tris-buffered saline, 0.1% Tween 20). Afterwards, the film was mildly washed with 1× TBST to remove any traces of skim milk left. Then the membrane was incubated with the respective primary antibodies. In each trial, the membranes were incubated along with horseradish peroxidase-conjugated anti-rabbit or mouse antibody and treated with enhanced chemiluminescence (ECL) detection reagents (PerkinElmer, ECL1:ECL2 = 1:1, Spokane, WA, USA) and used the Mini Size chemilluminecent Imaging System purchased from Life Science (St Petersburg, FL, USA) in order to detect bands.

### 4.10. Collagen Measurements

To compare the collagen created by fibroblasts with the testing sample incubated on 24-well plates with the total amount of collagen produced by fibroblasts, the latter collagen was stained with Sirius Red dye (direct Red; Sigma-Aldrich, St. Louis, MO, USA) [25,26]. The mediums were removed from the test subject after a pre-determined time interval and the cells were washed twice with PBS. The wells had an addition of 100 µL of 0.1% Sirius Red stain for 1 h at room temperature. The unattached stain from the well was removed and washed at least five times with 200 µL of 0.1 N HCl. The attached stain was extracted and thoroughly mixed with 100 µL of 0.1 N NaOH. The stain was then places into a 96-well plate in order to use a spectrometer at 540 nm (UV-VIS, BioTek, Winooski, VT, USA) to read the absorbance. The collagen amount produced per fibroblast cell was used in order to explain the specific cell collagen production.

### 4.11. VEGF Secretion Assays

To conclude the VEGF, or vascular endothelial growth factor, produced after the human dermal fibroblasts were exposed to the testing compounds, an enzymelinked immunosorbent assay (ELISA) was used. The experimented dermal cells were placed under a cultured conditioned medium within 6-well plates. After 24 h of incubation, the supernatant was collected. The DuoSet ELISA development kits (R & D Systems, Minneapolis, MN, USA) allowed for the determination of VEGF amount secreted in the culture medium (OD_540_). The assay was conducted through the manufacturer’s instructions and the protein amounts were measured with pictogram per milliliter.

### 4.12. Statistical Analysis

Extract bio-functional assays in each platform were carried out in triplicate. The results are expressed as the mean ± standard deviation. Analysis of variance data was used for data analysis. *p* < 0.05 was considered to be statistically significant.

## 5. Conclusions

Taken together, our present work demonstrated the proliferation effect of EAE on human dermal fibroblasts via up-regulation of VEGF. EAE also enhanced TIMP1, which subsequently reduced the MMP proteins, increasing the amount of collagen. EAE could be a human skin cell growth enhancer, and our present work shed light on the molecular mechanism for wound repair.

## Figures and Tables

**Table 1:** Antioxidative properties of enriched astaxanthin extract (EAE) at 10 μg/mL on various assay plates. (-), no testing; DPPH: 1,1-Diphenyl-2-Picrylhydrazyl; EDTA: Ethylenediaminetetraacetic acid; BHA: Butylated hydroxyanisole.

Samples	Anti-Oxidative Properties		
	DPPH (%)	Chelating (%)	Reducing Power (OD700)
Vitamin C ^a^	86.5	-	-
EDTA ^b^	-	87.3	-
BHA ^c^	-	-	1.88 ± 0.03
EAE	45.3	54.7	1.17± 0.11

Data were expressed as a mean value of at least three independent experiments. ^a^ Vitamin C was used as a positive control on DPPH assay at 100 μM; ^b^ EDTA was used as a positive control on metal chelating ability at 100 μM; ^c^ BHA was used as a positive control on reducing power at 100 μM.

## Implications

Molecular mechanism Enriched astaxanthin extract has the ability to decrease MMP enzyme expression in human dermal fibroblasts to enhance collagen production.
